# Using Distributional Statistics to Acquire Morphophonological Alternations: Evidence from Production and Perception

**DOI:** 10.3389/fpsyg.2016.00540

**Published:** 2016-05-03

**Authors:** Helen Buckler, Paula Fikkert

**Affiliations:** ^1^Department of Psychology, University of Toronto Mississauga, MississaugaON, Canada; ^2^Centre for Language Studies, Radboud University NijmegenNijmegen, Netherlands

**Keywords:** first language acquisition, lexical representations, production, perception, alternations

## Abstract

Morphophonological alternations, such as the voicing alternation that arises in a morphological paradigm due to final-devoicing in Dutch, are notoriously difficult for children to acquire. This has previously been attributed to their unpredictability. In fact, the presence or absence of a voicing alternation is partly predictable if the phonological context of the word is taken into account, and adults have been shown to use this information (Ernestus and Baayen, 2003). This study investigates whether voicing alternations are predictable from the child’s input, and whether children can make use of this information. A corpus study of child-directed speech establishes that the likelihood of a stem-final obstruent alternating is somewhat predictable on the basis of the phonological properties of the stem. In Experiment 1 Dutch 3-year-olds’ production accuracy in a plural-elicitation task is shown to be sensitive to the distributional statistics. However, distributional properties do not play a role in children’s sensitivity to mispronunciations of voicing in a Preferential Looking Task in Experiment 2.

## Introduction

Non-allophonic morphophonological alternations, such as the Dutch voicing alternation (e.g., the final [t] of [bεt] *bed* ‘bed’ is voiced in its plural form, *bedden* [bεdən]), are difficult for children to acquire, in part because they are unpredictable (Van Wijk, 2007; Van de Vijver and Baer-Henney, 2011; Zamuner et al., 2011). However, many studies show that distributional statistics, the likelihood of segments occurring in a given sequence, play a role in (early) language acquisition and processing (e.g., Jusczyk et al., 1993, 1994; Mattys and Jusczyk, 2001). In this paper we use corpus data to investigate whether there are regularities in the distribution of the voicing alternation in Dutch child-directed speech, that is, whether voicing alternations are more or less frequent in a given phonological context, and experimental data to test whether these regularities play a role in children’s ability to establish accurate lexical representations of voicing alternations in morphological paradigms. Both production and perception data are presented.

The voicing alternation of interest appears within morphological paradigms in Dutch. The language has a two-way voicing contrast, which is apparent in the plural forms *bedden* ‘beds’ [b𝜀dən] and *petten* ‘caps’ [pεtən]. The voicing contrast is neutralized syllable-finally, and only voiceless obstruents are permitted in this position. The singular forms of these two example words are therefore *bed* [bεt] and *pet* [pεt]. Voicing alternations do not occur in complementary distribution, and not all morphological paradigms contain voicing alternations. Because of this, children have great difficulty in learning which paradigms contain a voicing alternation and which do not (Peperkamp and Dupoux, 2002; Pierrehumbert, 2003).

Evidence from production and comprehension data highlight the difficulty that voicing alternations pose for children (Kerkhoff and De Bree, 2005; Kerkhoff, 2007; Van Wijk, 2007; Van de Vijver and Baer-Henney, 2011; Zamuner et al., 2011; Buckler and Fikkert, 2015). In a plural elicitation task, Kerkhoff (2007) found that at 7 years of age children achieve only 57% accuracy in their productions of plurals with a voicing alternation. In 41% of their responses they produced a devoicing error, e.g., *bedden* ‘beds’ as ^∗^[bεtən]. Conversely, they made voicing errors in only 2% of their responses for non-alternating words, e.g., *petten* ‘caps’ as ^∗^[pεdən]. Younger children at 3–5 years of age also participated in this study and their accuracy scores were even lower than those of the 7-year-olds. A possible explanation for children’s difficulty may be articulatory in nature: they do not have the ability to reliably produce a voicing contrast in medial position. This does not seem to be the case though, given that Zamuner et al. (2011) demonstrated in an imitation task that 3-year-olds are able to produce both [t] and [d] word-medially. A more likely explanation is representational, and children simply do not yet have a reliable representation in their mental lexicon of whether a voicing alternation occurs in a paradigm or not, despite the knowledge that the plural can be formed by suffixing *-en* to the singular form.

A recent study by Buckler and Fikkert (2015) provides evidence that children’s production errors derive from immature representations. Using a preferential looking paradigm (cf. Swingley and Aslin, 2000), they tested Dutch 3-year-olds’ sensitivity to mispronunciations of voicing in plural words (e.g., *petten* ‘caps’ would be pronounced *^∗^pedden* ‘caps,’ or *bedden* ‘beds’ as *^∗^betten*). Their results indicate that Dutch toddlers do not have robust representations of voicing in familiar plural words. The authors further compared Dutch and German toddlers in this task, as German displays a similar pattern of voicing alternation. They found that German children have more robust representations than their age-matched Dutch peers, and were more sensitive to voicing mispronunciations in this context. The authors argue that language-specific factors are driving this difference. Specifically, the voicing contrast has a higher functional load in German and there are more words containing voicing alternations. As such, German children are confronted with more evidence for voicing alternations in their language, assisting them in forming representations of voicing alternations within morphological paradigms. A further difference that may be contributing to German children’s enhanced performance in this task relates to the stimuli used in each language. The Dutch target words all contained a vowel immediately before the obstruent that is mispronounced, that is, words like *bedden* ‘beds’ and *petten* ‘caps.’ The German items used contained words with preceding vowels (e.g., *Betten* ‘beds’) and sonorants (e.g., *Hunde* ‘dogs’). In the current paper we investigate the possibility that distributional statistics reflect the likelihood that a voicing alternation is required in the morphological paradigm or not. Given that it is claimed that the unpredictability of voicing alternations is one of the primary reasons that children have so many difficulties acquiring the system (Peperkamp and Dupoux, 2002; Pierrehumbert, 2003), here, we test whether there is in fact some predictability in the system, and whether Dutch-learning 3-year-olds are sensitive to this information.

Previous studies with adults indicate that voicing alternations are not completely unpredictable in Dutch. In a corpus of adult speech, Ernestus and Baayen (2003) found that the likelihood of a voicing alternation occurring within a morphological paradigm is partly predictable if the final and penultimate segments of the stem are known. Of stems with a final voiceless segment, their data indicate that fricatives are more likely to alternate than plosives. For example, a stem-final [t] is likely to remain [t] throughout the paradigm (e.g., *pet-petten* ‘cap(s)’), but a stem-final [f] is likely to become [v] in the inflected form (e.g., *duif-duiven* ‘pigeon(s)’). If the phonological properties of the preceding segment are taken into consideration, the likelihood of an alternation occurring becomes even more predictable. Stems with a final obstruent are more likely to contain an alternation in the morphological paradigm if the penultimate segment in the stem is a sonorant than if it is a vowel. In words with a penultimate sonorant, e.g., *eend* ‘duck,’ alternations are found in 70% of paradigms. That is, *eenden* ‘ducks’ conforms to the dominant pattern, and *tenten* ‘tents’ the non-dominant pattern. If the penultimate segment is a vowel, e.g., *bed* ‘bed,’ only 25% of paradigms contain an alternation. In this case, *petten* ‘caps’ conforms to the dominant pattern, and *bedden* ‘beds’ the non-dominant pattern. Adults make use of these distributional likelihoods to guide whether they produce an alternation or not. When asked to inflect novel words, their responses reflect the distributional statistics of the language (Ernestus and Baayen, 2003, 2004). We aim to establish whether children are also sensitive to distributional statistics and the likelihood of an alternation occurring in a morphological paradigm.

Infants and children are known to be sensitive to distributional statistics. From a young age they make use of this information to help them learn about their native language. For example, by 9 months of age, infants are sensitive to which combinations of sounds are legal in their ambient language, and to the frequency of different phoneme co-occurrences (Jusczyk et al., 1993). Infants prefer to listen to non-words containing highly probable sound combinations rather than sequences with less likely sound combinations (Jusczyk et al., 1994), and can use this information to help segment words from speech (Mattys et al., 1999; Mattys and Jusczyk, 2001). In word learning tasks both children and adults are able to learn, retain and repeat items with high phonotactic probabilities faster and with greater accuracy than items with low phonotactic probabilities (Vitevitch et al., 1997; Frisch et al., 2000; Storkel and Rogers, 2000; Treiman et al., 2000; Munson, 2001; Storkel, 2001; Hollich et al., 2002; Zamuner et al., 2004). In addition, when phonological alternations occur in patterns of complementary distribution, such as allophonic variation, infants are able to track probabilities and rapidly learn the alternating pattern (White et al., 2008; Seidl et al., 2009).

Thus, from previous work we can conclude that voicing alternations in the Dutch lexicon are not entirely unpredictable, and adults are sensitive to distributional statistics that predict whether an alternation is more or less likely to occur (Ernestus and Baayen, 2003). Furthermore, a wealth of studies show that infants and young children are remarkably good at using distributional information to learn about statistical regularities in their language. We hypothesized, therefore, that Dutch children would be able to make use of distributional information when learning about voicing alternations in their language. First, we report the results of a corpus study of Dutch child-directed speech, where we establish that similar distributional patterns are present in the child’s lexicon and adult language. We then report on results from two experimental studies. The first is a plural elicitation task with Dutch 3-year-olds, where participants were required to inflect words where we manipulated the distributional likelihood of an alternation being required or not by presenting words where the pre-final segment was a vowel or a sonorant. In the second experiment we use a preferential looking task to examine Dutch 3-year-olds’ sensitivity to mispronunciations of voicing in familiar words. We tested words in which a sonorant precedes the stem-final obstruent (e.g., *tenten* ‘tents’ and *eenden* ‘ducks’), and compare our results to those reported in Buckler and Fikkert (2015), where the preceding segment was always a vowel (e.g., *petten* ‘caps’ and *bedden* ‘beds’).

The focus is on 3-year-olds, as this is an age where Dutch children are competent users of plural morphology (Schaerlaekens, 1977; Zonneveld, 2004; Van Wijk, 2007), but make frequent errors in voicing alternations (Kerkhoff, 2007; Zamuner et al., 2011). The reported age of acquisition of the plural in Dutch is in line with the acquisition of the plural in other languages in both production (Cazden, 1968; de Villiers and de Villiers, 1972; Park, 1978; Berman, 1981; Mervis and Johnson, 1991; Bittner and Köpke, 2001; Raymond et al., 2008; Ravid and Schiff, 2009) and perception (Kouider et al., 2006; Jolly and Plunkett, 2008). Despite using and comprehending plural morphology, Dutch children’s productions of voicing alternations in frequently occurring, familiar words are often inaccurate (Kerkhoff and De Bree, 2005; Kerkhoff, 2007; Zamuner et al., 2011). Because children are not expected to perform at ceiling level, we are able to examine whether accuracy is mediated by distributional statistics. Furthermore, comparing children’s knowledge of alternations in both production and perception provides insight into the two mechanisms and their interplay in the child’s lexicon.

### Corpus Analysis of Voicing Alternations in Child-Directed Speech

We conducted an analysis of Dutch child-directed speech comparable to the analysis of Ernestus and Baayen (2003) and found similar distributional patterns of alternating and non-alternating stem-final obstruents as attested in adult language. This indicates that children’s input provides them with information about the likelihood of voicing alternations occurring in different phonological contexts.

Through the CHILDES database (MacWhinney, 2000) we accessed all transcripts within the CLPF (Fikkert, 1994; Levelt, 1994) and Van Kampen corpora (Van Kampen, 1994) where the child was 3;6 or younger.^1^ We extracted all nouns for which there was both a singular and plural token in the corpus where the stem had a final plosive and the plural is formed with the suffix *-en*. Although, there are two productive plural suffixes in Dutch, *-en* and *-s*, we only considered *-en* in our analysis. The choice of suffix in Dutch is largely phonologically driven (Booij, 1995), with *-en* preferred following an obstruent or diphthong, or if the stem has final stress. The *-s* suffix is preferred if the stems ends in a vowel or unstressed syllable (see Booij, 1995 for more details). As only *-en* triggers voicing alternations, we excluded stems that take *-s* from our analysis. The corpus analysis was restricted to stem-final coronal and labial plosives because these segments provide the most reliable source of information about voicing in Dutch. The velar plosive is not informative as /g/ is not a native phoneme, therefore there is no [k]∼[g] alternation. Fricatives, belonging to the class of obstruents, should also be a source of information about voicing alternations, but fricative voicing is unreliable in Dutch and for speakers in many parts of the Netherlands the voicing contrast has been neutralized (van de Velde et al., 1996; Ernestus, 2000).

Overall type and token frequencies of items in our corpus are presented in **Table 1**. Reported token counts in this analysis refer to the plural form, as this form provides information about voicing alternations. Chi-square tests revealed a marginally significant trend in the distribution of voicing alternations by type frequency, χ^2^(1, *N* = 57) = 2.96, *p* = 0.085, but token frequency distribution did significantly differ from chance χ^2^(1,493) = 63.55, *p* < 0.05. This indicates that there are fewer singular–plural pairs that contain a voicing alternation. That is, *petten* ‘caps’ or *tenten* ‘tents’ are the more frequent pattern compared to *bedden* ‘beds’ or *eenden* ‘ducks.’

**Table 1:** Type and token frequency counts of alternations in singular–plural pairs.

Type frequency			Token frequency		
Total	Alternating	Non-alternating	Total	Alternating	Non-alternating
57	22 (38.6%)	35 (61.4%)	493	158 (32%)	335 (68%)

*All forms have a stem-final [t] or [p] and take the plural suffix -en. Token frequency counts refer to the frequency of the plural form, as this is where information about alternations is found.*

**Table 2** compares the frequency distribution of voicing alternations in our corpus of child-directed speech based on the phonological properties of the preceding segment. Due to there being very few words where the stem-final obstruent is preceded by an obstruent, we compared the distribution of voicing alternations following a vowel or sonorant consonant. For both type and token frequency there is a significant relationship between the phonological properties of the pre-final segment of the stem and whether or not an alternation occurs [Type, χ^2^(1,53) = 6.11, *p* < 0.05; Token, χ^2^(1,451) = 169.61, *p* < 0.05]. Voicing alternations are less likely in paradigms where the penultimate segment of the stem is a vowel than a sonorant. Of words with a pre-final vowel, no alternation in the plural is more frequent than an alternation, e.g., *bedden* ‘beds.’ However, the pattern is reversed in words with a pre-final sonorant obstruent, where alternations (e.g., *eenden* ‘ducks’) are more frequent than no alternations (e.g., *tenten* ‘tents’).

**Table 2:** Distribution of alternations in singular–plural pairs broken down by the phonological properties of the preceding segment.

	Type frequency			Token frequency		
Preceding segment	Total	Alternating	Non-alternating	Total	Alternating	Non-alternating
Vowel	30	8 (26.7%)	22 (73.3%)	279	34 (12.2%)	245 (87.8%)
Sonorant	24	14 (58.3%)	10 (41.7%)	177	124 (70%)	53 (30%)
Obstruent	3	0	3 (100%)	37	0	37 (100%)

*Token frequency counts refer to the frequency of the plural form.*

The pattern of alternations by phoneme sequence in the stem in our corpus of child-directed speech is comparable to the distribution attested in a corpus of adult speech (Ernestus and Baayen, 2003). We predict that Dutch-learning children will make use of this information when learning about voicing alternations in their lexicon. In terms of production accuracy, which is tested in a plural elicitation task in Experiment 1, we predict that toddlers will be more accurate in producing alternations in words with a pre-final sonorant in the stem than a pre-final vowel. That is, their production of *eenden* ‘ducks’ will be more accurate than their production of *bedden* ‘beds.’

Production studies provide only limited insight into the representations stored in the mental lexicon. The role of articulatory control is important, and at a representational level the relationship between children’s productive and receptive lexical representations is not yet well understood. As Swingley and Aslin (2000) discuss, the literature reveals a number of examples of children knowing more than they can say, and production data frequently underestimates children’s abilities. The classic example is the *sip-ship* case (Smith, 1973), where the child says *sip* for *ship*, but rejects this form if produced by an adult. For these reasons, in Experiment 2 we test children’s sensitivity to mispronunciations of voicing word-medially in a preferential looking task. Results are compared to those published as Experiment 1 in Buckler and Fikkert (2015), and we predict that children will be more sensitive to mispronunciations of voicing in a post-sonorant context than a post-vocalic context.

## Experiment 1

Experiment 1 reports data from a production task with Dutch 3-year-olds. In this task participants played a picture-matching game with the experimenter; the child would take a picture card out of a bag, find the card with the matching picture, and label the pictures on the cards. Target words/pictures had a stem-final [t], half of which required an alternation in the plural (e.g., *bed-bedden* ‘bed(s)’), and half of which did not (e.g., *pet-petten* ‘cap(s)’). Phonological context was a between-subject factor, and children were randomly assigned to the post-vocalic or post-sonorant condition. We opted for a between-subject design so that we could elicit a number of different words with the same phonological properties of interest from each participant. Given the variable nature of children’s productions, their different familiarity with target words, and the number of trials we could expect them to participate in, using a within-subject design would have allowed us to elicit fewer word types in each context, increasing the likelihood of missing data points for a given participant in a given context. Target words in the post-vocalic condition ended in a stem-final vowel-[t] sequence (e.g., *pet* or *bed* ‘cap’ or ‘bed’), and target words in the post-sonorant condition had a sonorant, either /n/ or /r/, preceding the [t] (e.g., *tent* or *eend* ‘tent’ or ‘duck’). The measure of interest was whether children produced a voiced or voiceless obstruent in the plural, and whether accuracy was mediated by phonological context. In line with previous literature, we predicted that children’s productions would be more accurate in words where no alternation is required (e.g., Kerkhoff and De Bree, 2005; Kerkhoff, 2007; Zamuner et al., 2011). We also predicted that they would be more accurate in producing voicing alternations in a post-sonorant than post-vocalic context.

### Materials and Methods

#### Participants

Seventy nine children participated in the study, and data from 49 children were included in the analysis (*M* age = 37 months, 28 days, range = 36 months, 29 days – 38 months, 25 days, female = 24). Data from 27 3-year-olds were included in the post-vocalic condition (*M* age: 37 months and 28 days, range = 37 months, 7 days – 38 months, 25 days, female = 14) and data from 22 children were included in the post-sonorant condition (*M* age: 38 months and 2 days, range = 37 months, 7 days – 38 months, 14 days, female = 10). Data from 30 children (post-vocalic condition = 13, post-sonorant condition = 17) were removed from the analysis because they did not produce a minimum of one token of both an alternating and non-alternating word of sufficient quality to be acoustically analyzed after exclusion criteria had been applied (*n* = 28), or from lack of data due to a technical error (*n* = 2; see Data Analysis below for details of exclusion criteria). Children were recruited through the Baby Research Center of the Max Planck Institute for Psycholinguistics and Radboud University Nijmegen. Ethical approval was obtained from the *Ethiek commissie faculteit der Sociale Wetenschappen (ECSW)* at the Radboud University Nijmegen.

#### Materials

The stimuli consisted of 16 nouns with a stem-final [t] that take the plural suffix *-en.* The stimuli set is presented in **Table 3**. Half of the nouns had a sonorant preceding the stem-final obstruent, and half a vowel. Half of the nouns contained a voicing alternation in the plural, and half did not. The words with a post-vocalic stem-final [t] are the same items as used in Experiment 1 of Buckler and Fikkert (2015). As phonological context was a between-subject factor, each participant had eight target nouns to label. All of these contained either a post-vocalic stem-final [t] or a post-sonorant stem-final [t]. Half of the nouns in each phonological context required an alternation in the plural. The following criteria were used to select target words: (1) they should be easily depictable; (2) they should be familiar to children of this age; (3) targets should have a higher token frequency in the singular than the plural.

**Table 3:** Target items used in Experiment 1.

Word Type	Item		Gloss	CELEX sg. frequency	Yoked distractor	Distractor gloss
Post-vocalic [t]	botten	[bɔtən]	bones	314	bomen	trees
	fluiten	[floeytən]	flutes	201	fietsen	bikes
	noten	[no:tən]	nuts	379	neuzen	noses
	petten	[pɛtən]	caps	698	peren	pears
Post-vocalic [d]	bedden	[bɛdən]	beds	12052	boeken	books
	broden	[bro:dən]	breads	2616	brillen	glasses
	hoeden	[hu:dən]	hats	1314	handen	hands
	kleden	[kle:dən]	rugs	455	klokken	clocks
Post-sonorant [t]	kaarten	[ka:rtən]	maps	3742	kaarsen	candles
	olifanten	[olifɑntən]	elephants	428	ooievaars	storks
	taarten	[ta:rtən]	cakes	437	tafels	tables
	tenten	[tɛntən]	tents	1141	tenen	toes
Post-sonorant [d]	eenden	[endən]	ducks	1013	eekhorns	squirrels
	manden	[mɑndən]	baskets	827	manen	moons
	paarden	[pa:rdən]	horses	6675	poezen	cats
	zwaarden	[zwa:rdən]	swords	650	zwembandjes	water wings

*Yoked distractors are relevant for Experiment 2. CELEX frequency reported is for the singular form of the word.*

Criterion 2 is typically addressed by selecting items that appear in standardized vocabulary lists, such as the Dutch version of the MacArthur Communicative Development Inventory (Zink and Lejaegere, 2002). This list, however, does not contain information about inflected forms of specific words, so we also considered frequency of occurrence of inflected forms in corpora of children’s speech. Using the CHILDES database (MacWhinney, 2000) we accessed all transcripts from the CLPF (Fikkert, 1994; Levelt, 1994) and Van Kampen (1994) corpora where the child was under 3;6. These were the same transcripts as studied in the corpus analysis reported in the previous section, but here we included the child’s utterances in addition to the child directed speech. We assumed that if a word appears in the corpus it is likely to be at least minimally familiar to the 3-year-olds participating in our experiment. In addition to selecting items that should be familiar to all children, we also used parental reports to gauge individual children’s familiarity to each item. One week before participating in the experiment parents were sent a picture book and accompanying questionnaire. The book contained 64 color images and the orthographic form of the intended referent. All items appeared in either Experiment 1 or 2 as targets, distractors or fillers, and the images were the same color photographs that would be used during the experiments. In the book all items were presented in the singular form to avoid drawing attention to the experimental question, and because we were interested in whether the target items formed part of the child’s lexicon or not. Parents were asked to read the book together with their child and indicate, in a similar manner to the MacArthur Communicative Development Inventory (Fenson et al., 1993), which words their child said, and which words they understood but did not produce. In addition, we asked them to indicate whether their child recognized the image as its intended referent. If a parent indicated that their child produced, comprehended or recognized the picture as its intended referent we concluded that the word was familiar to the child. If a word was reported as being unfamiliar to a child we removed that token from the analysis. Parental reports indicated that we successfully selected targets that were familiar to the majority of children of this age. Of the 49 children whose data was analyzed in both conditions, 31 trials (out of 312) were excluded due to the child being unfamiliar with the target word. The 27 participants included in the post-vocalic condition were familiar with an average of 3.8 (out of 4) items in the requiring an alternation, and 3.37 items that did not require an alternation. For the 22 participants whose data was analyzed in the post-sonorant condition mean familiarity was 3.59 for alternating words and 3.55 for non-alternating words.

Criterion 3, that the singular should be more frequent than the plural, ensured that children should be aware of the morphological link between singular and plural forms, and that the plural form is morphologically complex. It has been hypothesized that children do not interpret highly frequent plurals, for example *tanden* ‘teeth,’ as morphologically complex, but instead treat them as non-decomposable units (cf. Tesar and Prince, 2003). Frequency counts were obtained from the CELEX database, accessed via the Reetz-CELEX interface (Baayen et al., 1993; Reetz, 2010). Two items in the post-vocalic condition, *noten* ‘nuts’ and *botten* ‘bones,’ violated this criterion but were nonetheless included as they fulfilled all the other criteria better than other possible items. Furthermore, the item *botten* has two related meanings depending on the context or audience. The more frequent usage in adult language refers to the bones of the skeleton, whereas the child’s use of the word refers to a dog’s bone. This difference was apparent in the CHILDES frequency count, where the singular form was more frequent than the plural.

One image of the target item was printed on the center of a card approximately 10 cm square and laminated. All images were color photographs printed on a gray background. There were two identical cards per item. A small piece of Velcro was affixed to the reverse of each card allowing them to adhere to a soft surface. Per condition there were 14 pairs of cards: eight were test items and six were filler items. The filler items were *auto* (‘car’), *bal* (‘ball’), *hand* (‘hand’), *oog* (‘eye’), *poes* (‘cat’), and *sleutel* (‘key’). Filler items were the same for both the post-vocalic and post-sonorant condition, with the exception that *hand* was replaced by *kikker* (‘frog’) in the post-sonorant condition as *hand* contains the target context. These items occur in the earliest lists of words learned by children, and 3-year-olds should have little difficulty in labeling them in both the singular and plural.

#### Procedure

Prior to the start of the experiment one card from each pair was attached to a freestanding board in a grid pattern at a height accessible to a child. The remaining cards were placed in a small drawstring bag. During the experiment the experimenter sat or knelt on the floor and the child stood. A digital voice recorder (Olympus WS-650S) was placed on the floor at the base of the board. The child was instructed to take a card from the bag, find the matching picture on the board and hang their card next to it. They were encouraged to label the card whilst looking for the matching card, and once they had found the pair they were prompted to label the plural form, e.g., “Well done, now you have two....” Once the child had hung all pictures on the board they were asked to name the pairs once more.

#### Data Preparation and Analysis

Responses were recorded on a digital voice recorder and edited in *Praat* (Boersma and Weenink, 2011). All files were transcribed manually to identify the position of target words within the recording session. Plural target forms were extracted and the quality of the recording was judged as adequate or not. Due to the nature of the task a number of tokens had to be removed because noise masking the speech, for example, if the child jumped or pulled a card from the board while uttering the target word.

In this analysis we were specifically interested in the pronunciation of word-medial voicing, rather than the acquisition of plural morphology. For this reason we did not include tokens where the child produced a plural form that differed from our expected target, for example by producing a different lexical item or using the diminutive suffix, even if this was grammatically correct. These forms do not match the phonological context of interest, as an alternation never occurs in this position, therefore we did not include them in our analysis.

Accuracy of children’s productions of voicing was judged by three phonetically trained adult Dutch native-speakers who classified each token as containing a [t] or [d]. To reduce possible effects of lexical bias (cf. Ganong, 1980), adults were presented with only the medial VCV or Vson.CV spliced from the child’s production (e.g., *bedden* became *edde*). Targets were spliced to include 75% of the vowel duration, thereby providing enough information about the vowel quality but reducing lexical information that may be gained from co-articulation effects, for example, formant transitions between the initial segment and vowel. Coders listened to each token over closed headphones (Sennheiser HD 215) in a quiet room, and in a forced choice task indicated whether they heard a [t] or [d] in the children’s tokens. They also had to indicate, on a five-point scale, how sure they were of their response. All coders stated that they could not recognize the original lexical item from the VCV segment.

Because adult listeners are sensitive to phonological sequencing likelihoods, we were concerned that their lexical knowledge would bias them toward [d] judgments following a sonorant consonant and [t] following a vowel. As this would work in favor of our experimental hypothesis we needed ensure that the adults were able to make unbiased judgments. We recorded five (different) adult speakers producing all 16 target plural forms. Adults were presented with pictures of individual items and asked to produce the plural form. Recordings were made using the same recording device in a quiet office with a similar level of background noise to the Baby Research Center. Adult speakers were assumed to accurately produce [t] or [d] in each token. The medial VCV or Vson.CV segment was spliced out of each token in the same manner as the children’s tokens. The three phonetically trained listeners classified each segment as containing a [t] or [d] in the same way the classified the children’s tokens. Adult listeners’ ability to code the pronunciation of tokens produced by adult speakers was near perfect. Two of the three coders accurately classified voicing in all 80 of the adult tokens and the third made one error. These high accuracy scores indicated that coders could reliably base their decisions on the acoustic stimuli and were not influenced to lexical or other perceptual biases.

When coding the children’s productions, adult listeners were presented with 400 tokens from 58 participants to classify. 31 tokens were removed from the analysis because parents indicated these words were unfamiliar to their child. A further eight tokens from four participants were removed because these children did not contribute at least one alternating and one non-alternating token. If coders did not agree on the voicing value of a token, that token was removed from further analysis. Fifty-six tokens were removed for this reason. Disagreement was spread across coders, that is, no one coder consistently disagreed with the other two. A further 12 tokens from five participants had to be removed, as the child no longer contributed at least one alternating and one non-alternating token to the data set after all other exclusion criteria had been applied. There were 293 tokens from 49 participants remaining in the analysis of production accuracy. Each participant contributed an average of 6 tokens (range: 2–13), including both alternating and non-alternating forms. The data were balanced across conditions. In the post-vocalic condition participants contributed an average of 6.04 tokens, compared to 5.91 for participants in the post-sonorant condition. There were also a similar number of alternating and non-alternating tokens in the analysis, with participants contributing on average 2.79 alternating tokens and 3.24 non-alternating tokens.

The variable of interest in our data was children’s production accuracy in voicing of the medial /t/ or /d/. Data were analyzed using a mixed effect model, calculated using the *glmer* function of the *lme4* package (package version 1.1-10) in R Core Team (2012) and Bates et al. (2015). Predictors of production accuracy were Target Voicing (/d/ or /t/), Phonological Context (post-vocalic or post-sonorant) and the interaction of these two factors. The reference levels were voiced and post-vocalic. Random intercept terms were included for Subject and Item, and a random slope term was included for Subject by Target Voicing. This was the maximal random effects structure justified by the experimental design (Barr et al., 2013).

### Results

**FIGURE 1:**
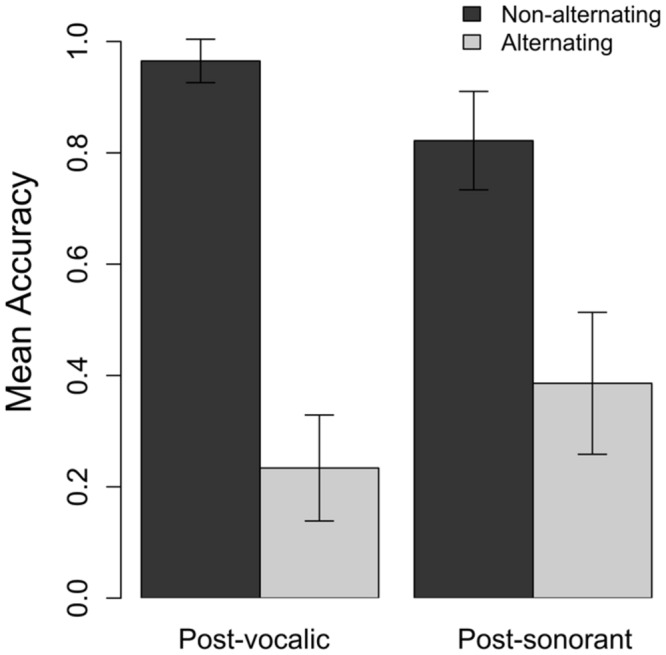
**Production accuracy in phonological context and target voicing in Experiment 1.** Accurate production of voicing in non-alternating targets indicates that the child correctly produced a [t] in the plural, e.g., *petten* ‘caps’ as the plural of *pet.* Accurate production in alternating forms indicated that they produced a voiced [d] where required, e.g., in the word *bedden* ‘beds.’

Accuracy results by target voicing and phonological context are presented in **Figure 1**. Overall, participants were accurate in their production of target voicing in 62% of tokens (183 out of 293). As predicted, both target voicing and phonological context affected production accuracy, and the interaction of these two factors. Model estimates are presented in **Table 4**.

**Table 4:** Regression model of production accuracy in Experiment 1.

	Estimate	*SE*	*z*-value	*p*-value
(Intercept)	-6.1	2.03	-3.0	0.003^∗∗^
Voicing (d vs. t)	16.19	4.86	3.33	<0.001^∗∗∗^
Phonological Context (vowel vs. sonorant)	4.79	2.39	2.01	0.045^∗^
Voicing ^∗^ Phonological Context	-8.5	4.04	-2.1	0.035^∗^

*^∗^*p* < 0.05, ^∗∗^*p* < 0.01, ^∗∗∗^*p* < 0.001.*

Children were more accurate in their production of target words that did not require an alternation, that is, words that have a [t] throughout the morphological paradigm (Target Voicing: β = 16.19; *SE* = 3.33; *p* < 0.001). In the post-sonorant condition children were more accurate in their production of alternating targets than they were in the post-vocalic condition (Phonological Context: β = 4.79; *SE* = 2.4; *p* < 0.05). The interaction of Target Voicing and Phonological context was significant (Target Voicing x Phonological Context: β = -8.5; *SE* = 4.04; *p* < 0.05). *Post hoc* pairwise comparisons were conducted on the model output using the function *glht* from the package *multcomp* (Hothorn et al., 2008). This package provides *z*-values and *p*-values corrected for multiple comparisons. Pairwise comparisons indicate that the effect of Target Voicing was greater in the post-vocalic context than the post-sonorant context. In the post-vocalic condition participants were significantly more accurate in their production of target words that do not require an alternation than with words that do; this is the effect of Target Voicing that was present in the original model. In the post-sonorant condition there was no significant difference in accuracy of words that did or did not require an alternation (β dif.: 7.69, *SE* = 3.99, *p* = 0.09).

In a final analysis we calculated whether there was a correlation between lexical frequency and production accuracy. Frequency counts were log transformed, and frequency counts of 0 were substituted with 0.5, as the natural log of 0 is undefined. There was no relationship between production accuracy and an item’s plural frequency in the CHILDES database (*r* = 0.14, *p* = 0.6), or the combined frequency of the singular and plural form in the CHILDES database (*r* = -0.1, *p* = 0.7). Similarly, there was no relationship between production accuracy and plural frequency in the CELEX database (*r* = -0.003, *p* = 0.99), or the combined singular and plural frequency (*r* = -0.36, *p* = 0.17).

### Discussion

As predicted, children were more accurate in their productions of word-medial voicing in plural forms when there was no voicing alternation between the stem and plural form. Another way of formulating this is to say that children made more devoicing errors in alternating forms than voicing errors in non-alternating forms. This result is in line with previous plural elicitation tasks with Dutch children (Kerkhoff and De Bree, 2005; Kerkhoff, 2007; Zamuner et al., 2011). We were particularly interested in how children’s accuracy of voicing in alternating words was affected by phonological context, and hypothesized that they would be more accurate and make fewer devoicing errors in a post-sonorant context than post-vocalic context. Our results indicate that children are paying attention to phonological context. As predicted on the basis of adult experimental data and child-directed speech corpus data, children produced a [d] more accurately in words where it is preceded by a sonorant than a vowel, indicating that they are sensitive to distributional statistics in determining whether a stem-final [t] alternates in the plural or not.

If children are sensitive to the frequency of alternations following a nasal, the reverse can also be predicted, that children may overapply their knowledge of phonological context and alternations. They may have a bias for post-nasal voicing and produce more voicing errors in non-alternating forms when preceded by a sonorant than by a vowel. This prediction is also upheld in our data; children had a mean accuracy score of 97% for post-vocalic, non-alternating words, and 82% for post-sonorant, non-alternating words. That is, voicing errors of the type *^∗^fluiden* (*fluiten*, ‘flutes’) occur in 4% of tokens, and of the type ^∗^*tenden* (*tenten* ‘tents’) in 18%.

A usage-based theory of language acquisition (e.g., Tomasello, 2003) would claim that children’s familiarity with a word guides their production accuracy, rather than the phonological context and sensitivity to segmental pattern frequencies. That is, if a word has been heard more often, children will have a more robust representation of its form and therefore produce it with greater accuracy. This explanation would predict a correlation between the lexical frequency and production accuracy. We find no such correlation in our data, indicating that lexical frequency is not driving children’s production accuracy of voicing in the plural forms tested here. It should be noted that children were only tested on a small number of tokens and frequency was not explicitly manipulated as a focus of the study, therefore the relationship between frequency and production accuracy can only be speculative.

A further contributor to our results could be ease of articulation. Articulatory accounts would predict that voiced obstruents are more likely to be perceived in both the post-vocalic and post-sonorant condition. Voicing through the closure is an important cue for detecting word-medial voicing (Slis and Cohen, 1969). In order to produce a voiceless obstruent the speaker must actively stop glottal fold vibrations. The relative timing of articulator movement may result in some degree of voicing remaining through the closure. Articulatory effects are exacerbated post-nasally, an environment where voicing is reported to be phonetically natural (cf. Kager, 1999). The lowered velum required for the nasal must be raised for the obstruent, but if the velum is not fully raised before the onset of the obstruent some air may flow through the nasal cavity. This “nasal leak” can be perceived as voicing (Hayes and Stivers, 1995). However, in a word-imitation task previous production data from Dutch 3-year-olds has revealed that they have little difficulty in producing word-medial voiced or voiceless segments, and actually produce voiceless segments more reliably than voiced obstruents (Zamuner et al., 2011). In our data, if articulatory difficulties were driving the attested effects, we would expect to see greater accuracy of voiced segments than voiceless segments, or at least substantial voicing errors of voiceless targets, which was not the case. Neijt and Schreuder (2007) argue although voiced segments are easier to produce word-medially, when uttered by a child they may be perceived as voiceless because of the slow speed of the child’s articulation. They claim that voiceless segments favor slow articulation, requiring longer periods of closure or aspiration than voiceless obstruents, and the slow speed of children’s articulations may extend a voiced obstruent to the extent that an adult perceives it as voiceless. Even though children’s articulation may be generally slower than that of adults, previous studies have shown that children of a similar age reliably make a voicing contrast in word-medial position and that adults can interpret this correctly (Kuijpers, 1993; Zamuner et al., 2011). Speech of young children is more variable than that of adults (e.g., Smith, 1978; Smith et al., 1983), making it possible that some tokens were misperceived by adult listeners, however, this is unlikely to be the primary reason for the [t]-bias in our data. Ease of articulation and perception undoubtedly contributed to our data somewhat, but they cannot explain our results entirely.

Thus, the results of Experiment 1 indicate that in Dutch, 3-year-olds’ production accuracy is sensitive to phonological sequencing and the likelihood of a voicing alternation occurring in a morphological paradigm. In Buckler and Fikkert (2015), Experiment 1, the authors use the Intermodal Preferential Looking Paradigm (Golinkoff et al., 1987) to test Dutch toddlers’ sensitivity to mispronunciations of voicing in plural forms (e.g., *petten* as *^∗^pedden* ‘caps’ or *bedden* as *^∗^betten* ‘beds’), thereby testing the representation of voicing in the toddlers’ receptive lexicon. This method provides insight into the phonetic specificity of representations stored in the mental lexicon. If the child is familiar with a word and has a detailed phonetic representation they will notice mispronunciations that deviate from their expectation, and this will be apparent in their gaze behavior. Buckler and Fikkert (2015) only tested words where the stem-final obstruent occurs in a post-vocalic context, and found that toddlers do not have a robust representation of voicing alternations in lexical items in this context. In Experiment 2 we extend the results of Buckler and Fikkert (2015) and test Dutch toddlers’ sensitivity to voicing in word-medial forms in a post-sonorant context, for example, *tenten* as *^∗^tenden* ‘tents’ or *eenden* as *^∗^eenten* ‘ducks.’

## Experiment 2

Experiment 2 reports data using the Intermodal Preferential Looking Paradigm (Golinkoff et al., 1987) in a task designed to measure children’s sensitivity to mispronunciations of voicing in familiar words (Swingley and Aslin, 2000). We tested whether children are sensitive to mispronunciations of voicing in word-medial position of familiar plural forms, for example, *tenten* pronounced as *^∗^tenden* (‘tents’) or *eenden* as *^∗^eenten* (‘ducks’). In addition to plural words, we also tested children’s sensitivity to mispronunciations of voicing in word-medial position in monomorphemic words, e.g., *wortel* as *^∗^wordel* ‘carrot’ or *vlinder* as *^∗^vlinter* ‘butterfly.’ In these items the mispronunciation occurs in the same phonological context as the plural words, but because it is not at a morpheme boundary – a potentially alternating position – we expected children to have a robust representation of voicing in these words.

When formulating hypotheses regarding how children will respond to mispronunciations in plural forms, there are a number of interacting factors that lead to different predictions depending on the relative importance of each factor in the child’s mental lexicon. The basic assumption of the paradigm is that if participants have a robust phonological representation of a lexical item they will be sensitive to mispronunciations in its form, and look less to the target image when its label is mispronounced compared to when it is correctly pronounced. The most general prediction, therefore, would be that children would notice all mispronunciations; they will be sensitive to mispronunciations of voicing in both directions, in both plural and monomorphemic words. Furthermore, phonological context would not play a role, and sensitivity to mispronunciations would not be affected by whether the preceding phoneme is a vowel or sonorant.

However, we predicted that this general sensitivity is influenced by phonological context. To assess the contribution of phonological context we compare our results here, which test sensitivity to mispronunciations in a post-sonorant context, to data presented in Experiment 1 of Buckler and Fikkert (2015), where children’s sensitivity to mispronunciations in a post-vocalic context were tested.

Based on the frequency of occurrence of voicing alternations in different phonological contexts, a pattern that was reflected in the production task in Experiment 1, two possible predictions can be made. On the one hand, children may have more robust representations of voicing alternations in a post-sonorant context than a post-vocalic context, and be more sensitive to mispronunciations in this task than in Experiment 1 of Buckler and Fikkert (2015). On the other hand, they might overgeneralize the likelihood of an alternation occurring based on their sensitivity to the distributional statistics of their language. In this case one would predict that children expect to hear a voicing alternation following a sonorant, and no voicing alternation following a vowel. In a post-sonorant context they would have a [d]-bias, regardless of what the correct pronunciation should be. That is, *eenden* and *^∗^tenden* would be preferred pronunciations of the words for ‘ducks’ and ‘tents,’ even though one is a mispronunciation. The reverse pattern would be predicted in a post-vocalic condition, namely, they would have a [t]-bias, preferring *petten* and *^∗^betten* for ‘caps’ and ‘beds,’ again, despite one being a mispronunciation.

Despite the results of Experiment 1 supporting the claim that children are sensitive to distributional statistics in producing voicing alternations, accuracy was not high. Even though production accuracy was mediated by phonological context, overall, children were much more likely to produce a [t] in a plural form than a [d]. If preference for [t] in plural forms is a reflection of children’s lexical knowledge, one could predict that children will have a bias toward target words presented with [t] in Experiment 2, and this bias might override any effect of phonological context.

When predicting behavior in Experiment 2 we also need to consider the pattern of results previously attested in Buckler and Fikkert (2015), who tested children’s sensitivity to mispronunciations of voicing in a post-vocalic context. In this context Buckler and Fikkert (2015) found that children display the opposite behavior and overgeneralize the voicing alternation. In words where no alternation is required they look more to the target when it is mispronounced than when it is correctly pronounced (e.g., *^∗^pedden* over *petten* ‘caps’). In words where an alternation is required they look less to the mispronounced target than to the correctly pronounced target (e.g., *bedden* over *^∗^betten* ‘beds’). If children have more robust representations of voicing alternations in a post-sonorant context, they may rely more on their representation rather than overgeneralizing, and may therefore not show a [d]-bias in the same way, but know the appropriate pronunciation and reject the mispronunciation.

### Materials and Methods

#### Participants

Thirty-nine children participated in this study. Data from 35 children were included in the analysis (mean age: 37 months and 28 days; range: 36 months and 29 days – 38 months and 17 days; 15 girls). A further four children participated but were excluded from the analysis for fussiness or not participating in at least 8 of the 16 test trials. These children also participated in the post-sonorant condition of Experiment 1. Children were recruited through the Baby Research Center of the Max Planck Institute for Psycholinguistics and Radboud University Nijmegen. Ethical approval was obtained from the *Ethiek commissie faculteit der Sociale Wetenschappen (ECSW)* at the Radboud University Nijmegen.

#### Materials

Stimuli consisted of 16 bisyllabic nouns with word-medial /t/ or /d/ following a sonorant consonant /n/, /r/, or /l/. Half were plural forms, and half were monomorphemic (singular) forms. Mispronunciations were created by changing the feature voicing value of the word-medial, e.g., *tenten* became *^∗^tenden* ‘tents’ and *eenden* became *^∗^eenten* ‘ducks’ The plural target words were the same items used in the post-sonorant condition of Experiment 1 (cf. **Table 3**), and monomorphemic forms were selected that adhered to the same criteria (**Table 5**). An additional criterion was included, namely that all mispronunciations should result in non-words.

**Table 5:** Monomorphemic target items used in Experiment 2 and yoked distractor items.

Word type	Item		Gloss	Yoked distractor	Distractor gloss
Post-sonorant [t]	groente	[xruntə]	vegetable	geld	money
	skelter	[skɛltər]	go-cart	skippybal	space hopper
	winter	[υıntər]	winter	windmolen	windmill
	wortel	[υɔrtəl]	carrot	worst	sausage
Post-sonorant [d]	aarde	[a:rdə]	earth	aardbei	strawberry
	panda	[pɑndɑ]	panda	papagaai	parrot
	vlinder	[flındər]	butterfly	vogel	bird
	zolder	[zɔldər]	attic	zomer	summer

Each target item was yoked with a distractor image. The onset of the distractor image was matched to that of the target word in order to delay participants’ ability to make a decision between the target and distractor until later in the word. The yoked distractor items were expected to be familiar to children of this age.

Audio stimuli were produced by a female Dutch speaker in a child-directed manner. Recordings were made in a sound-treated recording booth and digitized at a sampling rate of 44.1 kHz and resolution of 16 bits in Adobe Audition. Stimuli were edited using Praat (Boersma and Weenink, 2011).

The visual stimuli were photographs of objects on a gray background. Three adult native Dutch speakers verified that all images were typical exemplars of the labeled category as it would be understood by a young child. Plural images were the same images used in the post-sonorant condition of Experiment 1.

#### Procedure

During the experiment children sat on their caregiver’s lap 60 cm away from the screen in a dimly lit room. The caregiver wore closed headphones and listened to music interspersed with speech throughout the experiment to mask the auditory stimuli and minimize any potential influence on their child’s behavior. Auditory stimuli were presented via centrally located loudspeakers below the screen. Target images were presented side by side on the 17-inch TFT monitor of a Tobii T60 eye tracker. A thin black vertical line divided the screen in two, and each image was positioned centrally in one screen half. In plural trials the visual display consisted of two identical images side-by-side in the screen half. Stimuli were presented using the Tobii-Studio software (Tobii Technology, 2008). The test began with a nine-point calibration procedure. If all points were not calibrated in the first attempt, individual points were recalibrated a second time. The experiment began immediately after calibration.

Each child was presented with four blocks of eight trials. Half of the trials were test trials, and half were filler trials. Of the 16 test trials, the target was correctly pronounced in eight trials and mispronounced in the other eight. Filler items were assumed to be familiar to children of this age, and were always correctly pronounced. The presence of filler trials increased the ratio of correct pronunciation to mispronunciation trials to 3:1. Filler trials were not analyzed.

The child was presented with all 16 target items exactly once in either its correct or mispronounced form (eight plural items and eight monomorphemic items). No image or label was repeated. Thus, no child was presented with the same target item in both a correct and mispronounced form. Mispronunciations were balanced for direction (/t/ to [d] or vice versa) across all word classes. Six different versions of the stimuli were created, ensuring that all target items occurred equally as correctly pronounced and mispronounced trials across all participants.

Prior to each trial a fixation cross was displayed in the center of the screen for 500 ms. Target and distractor images were displayed on screen for 1600 ms before the child heard *kijk!* (“look!”). 900 ms later, or 2500 ms from the beginning of the trial, the target word was presented. The trial ended after a further 2500 ms.

#### Data Preparation and Analysis

A number of criteria were applied to ensure the data analyzed were a reliable reflection of the child’s lexical knowledge. Firstly, individual unreliable measurement points were removed. The eye tracker automatically assigns data points a validity score that quantifies the quality of gaze data recorded. Each data point measured is given a value between 0 and 4 indicating how certain the eye tracker is of its measurement. 0 indicates it is certain that the data is valid, and 4 that the data point is missing or definitely incorrect. We adhered to the manufacturer’s recommendation and removed data points with a validity code of two or higher from analysis (Tobii Technology, 2008). Therefore, data points were removed where the child was not looking to the screen or where tracking quality was poor.

Secondly, data from whole trials were removed if the child was not participating in the task at that moment. Trials were removed if the child did not look to the screen at all during the trial, if they did not look to both the target and distractor image during the first 2500 ms of the trial, or they did not look to either the target or distractor image for at least 100 ms during second 2500 ms of the trial, following the utterance of the target word.

Thirdly, trials were removed on the basis of parental report. As described in Experiment 1, prior to coming to the lab parents were sent a picture book and word list to complete, indicating their child’s familiarity with the words that would appear in the test. Trials were removed from the analyses if the child was unfamiliar with either the target or yoked distractor.

The final criterion applied was to remove the participant from further analysis if, following all exclusion criteria, there were fewer than 50% of test trials remaining for analysis (fewer than 8 out of 16 trials). Data from seven children were removed for this reason. On average each child contributed 12.6 trials, out of a possible 16, to the analysis (*SD* = 2.4, range = 8–16), balanced across different trial types; pronunciation (227 correctly pronounced trials, 214 mispronunciation trials), target voicing (219 trials with canonical /t/, 222 trials with canonical /d/), and morphology (244 plural trials, 197 monomorphemic trials).

Two areas of interest (AOIs) were defined in the display, corresponding to the left and right half of the screen. Each AOI covered half of the display minus a 10 pixel-wide vertical line down the center. For each trial one AOI corresponded to the target image, and the other to the distractor image. Each data point measured by the eye tracker (60 per second) was coded for whether they were looks to the target AOI or distractor AOI. Fixations falling within either of the AOIs were considered object fixations. The few fixations falling outside either AOI were regarded as off screen and not taken into consideration in the analysis.

Participants’ looks to the target image were analyzed over a time-window extending from 300 to 1300 ms after the onset of the target word. This window of analysis is the same as used in Buckler and Fikkert (2015), allowing for comparability of results across studies. Data were analyzed using Growth Curve Analysis (Singer and Willett, 2003), a multi-level modeling framework that uses orthogonal polynomials in hierarchically related submodels to capture changes in the data pattern over time (see Mirman et al., 2008 for details of this method as applied to eye tracking data). The Level 1 submodel uses third-order polynomials to capture the overall time course of fixation curves. The third-order polynomial was necessary to capture the S-shape of the curve. The Level 2 submodel captures how experimental manipulations modulate the Level 1 intercept and linear terms. Effects on the intercept term reflect changes in the average height of the curve, analogous to an average measure looks to target used in traditional analyses. Effects on the linear term reflect changes in the gradient of the slope.

The analysis was run in R Core Team (2012) using the lme4 package (Bates et al., 2015). Fixed effects were Pronunciation (correct or mispronounced), Morphology (plural or monomorphemic) and Target Voicing (canonical voiced or voiceless), and an interaction of these three effects. The reference levels were correct pronunciation, plural and underlyingly voiced (i.e., canonical pronunciation with /d/). We did not include effects of experimental manipulation on all Level 1 time terms as it is unclear how these should be interpreted (Mirman et al., 2008). Random effects for participants and items were included on all time terms, allowing for variation in height, slope and curvature of the data for individual participants and words. *Post hoc* pairwise comparisons to assess the effect of mispronunciation on each word type were conducted using the function glht from the package multcomp (Hothorn et al., 2008). This package performs multiple comparisons on the model, providing *z*-values and corrected *p-*values for each comparison.

### Results

The intercept term reflects the average height of the curve; effects on this term are interpreted as differences in the overall proportion of target fixations. The linear time term reflects the average gradient of the curve; effects on this term are interpreted as differences in the speed of gaze-shift to the target image.

**FIGURE 2:**
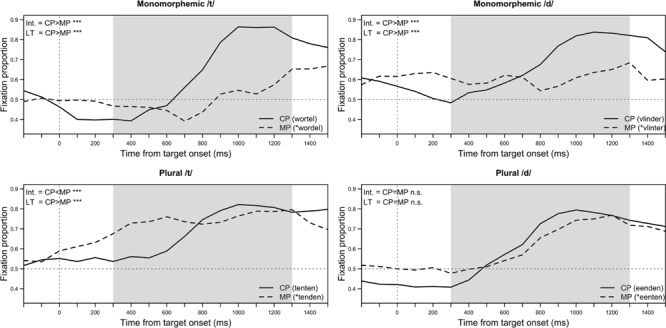
**Target fixations to different trial types in Experiment 2.** Solid lines represent looking behavior when the target word was correctly pronounced (CP), and dashed lines when the target was mispronounced (MP). The shaded area corresponds to the time-window of analysis, starting 300 ms after the target word onset for a duration of 1000 ms. The abbreviations ‘Int.’ and ‘LT’ stand for ‘Intercept’ and ‘Linear Time,’ and indicate statistical differences between the height (Intercept) and gradient (Linear Time) of the two lines in each panel. These statistical differences are summarized from the pairwise comparisons presented in **Table 6**.

Interactions in the model involving the factor Pronunciation are most relevant to the purposes of this study. The three-way interaction of Pronunciation, Morphology, and Target Voicing was significant on the intercept term (Intercept: β = -0.79, *SE* = 0.13, *p* < 0.001. Linear Time: β = -2.92, *SE* = 4.12, *n.s.*). The two-way interaction of Pronunciation and Morphology was significant on both the intercept and linear time term (Intercept: β = -0.24, *SE* = 0.09, *p* < 0.01. Linear Time: β = -14.56, *SE* = 2.83, *p* < 0.001), as was the interaction of Pronunciation and Voicing (Intercept: β = 0.41, *SE* = 0.9, *p* < 0.001. Linear Time: β = -9.16, *SE* = 2.71, *p* < 0.001). In addition, the effect of Pronunciation was significant on both the intercept and linear time terms (Intercept: β = -0.13, *SE* = 0.06, *p* < 0.05. Linear Time: β = -4.39, *SE* = 1.82, *p* < 0.05). Taken together, these significant effects and interactions all indicate that the effect of a mispronunciation on word recognition was significantly modified by the morphological status of the target word, as well as whether its canonical pronunciation should contain a /t/ or /d/. The effect of the mispronunciation is felt both on the overall looking time to the target and the speed of recognition. *Post hoc* pair-wise comparisons allow further investigation into the exact nature of the effect of pronunciation on different word types. Results of the pair-wise comparisons are presented in **Table 6**, and summarized in **Figure 2**, which shows the average target fixation to correct and mispronounced words.

**Table 6:** *Post hoc* comparison of the effect of Pronunciation on different word types in Experiment 2.

Morph.	Target voicing		CP Est.	MP Est.	Estimated dif. between CP and MP	*SE*	*z*-value	*p*-value
Plural	/t/	Int.	0.91	1.19	0.28	0.06	4.34	<0.001^∗∗∗^
		LT	22.24	8.69	-13.55	2.02	-6.71	<0.001^∗∗∗^
	/d/	Int.	0.65	0.52	-0.13	0.06	-2.3	0.16 *n.s.*
		LT	20.55	16.16	-4.39	1.82	-2.41	0.12 *n.s.*
Mono.	/t/	Int.	0.91	0.16	-0.75	0.07	-11.26	<0.001^∗∗∗^
		LT	34.19	3.16	-31.03	2.19	-14.15	<0.001^∗∗∗^
	/d/	Int.	0.86	0.49	-0.37	0.07	-5.4	<0.001^∗∗∗^
		LT	23.28	4.32	-18.96	2.18	-8.69	<0.001^∗∗∗^

*CP, Correct pronunciation; MP, mispronunciation; Int., intercept; LT, linear time. ^∗^*p* < 0.05, ^∗∗^*p* < 0.01, ^∗∗∗^*p* < 0.001.*

The effect of mispronunciations on gaze behavior in monomorphemic trials follows the pattern that would be expected if participants were sensitive to mispronunciations. For words with both a canonical medial /t/ or /d/ the intercept and linear time terms are higher for correct pronunciations than mispronunciations. That is, participants are faster to shift their gaze to the target image, and fixate it for longer, when the target word is correctly pronounced compared to when it is mispronounced.

The effect of mispronunciations on gaze behavior in plural trials is less straightforward. For plural words with a canonical /t/, that is, no voicing alternation within the morphological paradigm, participants display some sensitivity to voicing mispronunciations. They are faster to shift their gaze to the target image when it is correctly pronounced, reflected in the different estimates on the linear time term (LT β dif.: -13.55, *SE* = 2.02, *p* < 0.001). The difference in the intercept term is also significant, but in the opposite direction to expected: when the target is mispronounced, participants spent, on average, a longer time looking at the target image than when it was correctly pronounced (Int. β dif.: 0.28, *SE* = 0.06, *p* < 0.001). For plural words with a canonical /d/ pronunciation, that is, a voicing alternation within the morphological paradigm, we see no significant difference in looking behavior to correct or mispronounced trials. Dutch toddlers are sensitive to mispronunciations of voicing in plural forms when /t/ is presented as [d], but not when /d/ is presented as [t].

### Discussion

Results from Experiment 2 indicate that Dutch 3-year-olds are sensitive to mispronunciations of word-medial voicing in familiar words. Their looking behavior was significantly affected by mispronunciations of voicing in monomorphemic words, demonstrating that they were able to perceive voicing mispronunciations in this position, and that these have a detrimental effect on word recognition. This is true for mispronunciations in both directions, that is /d/ presented as [t] (e.g., *vlinder* as *^∗^vlinter* ‘butterfly’), and /t/ presented as [d] (e.g., *wortel* as *^∗^wordel* ‘carrot’). Because they are sensitive to mispronunciations in monomorphemic words, different patterns of looking behavior in plural trials can be attributed to the different morphological context and the possibility of a voicing alternation occurring, rather than a more general difficulty in noticing voicing mispronunciations in this phonological context. Toddlers displayed some sensitivity to mispronunciations of /t/ in plural words, and no sensitivity to mispronunciations of /d/. Target words were recognized when correctly pronounced for both plural /t/ and /d/ words, thus, we can assume that sensitivity, or lack thereof, to mispronunciations was not caused by their failure to recognize the target word.

With regard to mispronunciations of plural words, our results are somewhat consistent with an explanation that assumes children have overgeneralized the pattern that a voicing alternation should be present in the morphological paradigm if the stem-final obstruent is preceded by a sonorant consonant. For plural /t/ words, children looked, on average, more to the target when it was mispronounced than when it was correctly pronounced, possibly suggesting that they expect a [d], the mispronounced form, in this context. However, if this explanation were to hold entirely, we would also expect to see sensitivity to mispronunciations of voicing in plural /d/ words, with children looking less to the target when mispronounced (i.e., presented with [t]), than correctly pronounced. This pattern was not attested.

The primary question of interest was how phonological context affects children’s representations of voicing alternations, therefore the results of children’s looking behavior to plural trials in Experiment 2 are compared to the results of Buckler and Fikkert (2015) Experiment 1, where Dutch toddlers were tested on the same task but the stem-final obstruent was in post-vocalic position. Interestingly, Buckler and Fikkert (2015) also found that toddlers looked longer overall to mispronunciations of plural words with /t/, compared to their correct pronunciation (i.e., they looked more to *^∗^pedden* than *petten* ‘caps’). In addition, they found that toddlers displayed some sensitivity to mispronunciation in plural words with /d/, being faster to shift their gaze to the target when correctly pronounced than mispronounced (i.e., faster if *bedden* than *^∗^betten* ‘beds’). In a post-vocalic context, therefore, children have a bias toward expecting a voicing alternation in a plural form. In a post-sonorant context, children also have something of a bias toward expecting a voicing alternation in the plural form, although not as strong a bias as in Buckler and Fikkert (2015). The similarity of results across the two experiments is inconsistent with our hypothesis that sensitivity to distributional statistics and phonological context guides children’s ability to form lexical representations of plural words. It is not the case that they have more robust representations of alternations in a post-sonorant context than a post-vocalic context. In all cases it seems that Dutch 3-year-olds have some knowledge that voicing alternations may occur, but are not yet certain, at least for the lexical items in this stimulus set, which items require an alternation and which do not.

## General Discussion

The purpose of this study was to investigate the effect of distributional statistics on Dutch toddlers’ representations of voicing alternations in morphological paradigms. Previous research has indicated that the pattern of voicing alternations is difficult for children to learn and use reliably (Kerkhoff and De Bree, 2005; Zamuner et al., 2011; Buckler and Fikkert, 2015), in part due to the unpredictability of the pattern (Peperkamp and Dupoux, 2002; Pierrehumbert, 2003). However, corpus data had previously shown that the system is not entirely unpredictable, and if phonological context is taken into account there is actually a large degree of predictability that adults are able to make use of in cases where they are uncertain of what the correct form should be, for example in novel or unfamiliar words (Ernestus and Baayen, 2003). The current study comprised three parts. Firstly, we conducted a corpus study of Dutch child-directed speech, and established that similar distributional patterns are present in the early lexicon as the adults’ lexicon. Secondly, in a plural elicitation task in Experiment 1, we found that production accuracy of voicing in plural forms is sensitive to distributional statistics. Finally, in a mispronunciation detection task designed to test the specificity of lexical representations without the performance constraints of a production task, we find that children’s sensitivity to mispronunciations of voicing is not affected by phonological context. Thus, the initial prediction that children may be able to use phonological context and distributional statistics is to some extent supported by our data, but not entirely. A number of interesting questions arise about the difference between production and perception and developing lexical representations.

The results of Experiment 1 confirm our hypothesis that the Dutch language provides the child with statistical information about the likelihood of an alternation occurring in a morphological paradigm on the basis of the phonological structure of the stem, and that toddlers are sensitive to these distributions. Children’s production accuracy reflects the statistics, although it should be noted that their behavior is far from adult-like. Production accuracy was generally low in plural tokens where a voicing alternation was required; that is, participants were more likely to produce a voiceless segment than a voiced one. Failure to produce alternations in plural forms is consistent with previous findings in similar tasks (Kerkhoff and De Bree, 2005; Zamuner et al., 2011). Nonetheless, accuracy was mediated by phonological context, and children were more accurate in producing an alternation in a post-sonorant context than a post-vocalic context. This result is consistent with previous studies demonstrating that infants and children are sensitive to the phonotactic probabilities of their ambient language (Jusczyk et al., 1993, 1994; Vitevitch et al., 1997; Mattys et al., 1999; Storkel and Rogers, 2000; Treiman et al., 2000; Mattys and Jusczyk, 2001; Munson, 2001; Storkel, 2001; Hollich et al., 2002; Coady and Aslin, 2004; Zamuner et al., 2004). In addition, White et al. (2008) and Seidl et al. (2009) have shown that infants can use allophonic variation to learn alternating patterns. Voicing alternations are not allophonic in Dutch, but morphophonemic; alternations do not occur in complementary distribution, making it more difficult to form generalizations, but our data show that children are sensitive to distributional patterns.

Given that children’s production accuracy shows sensitivity to phonological context and distributional statistics it is surprising that the results of Experiment 2, when compared to the results of Experiment 1 of Buckler and Fikkert (2015), do not also reflect this result. In many aspects of language acquisition it is often reported that there is an asymmetry between children’s comprehension and production abilities. However, the asymmetry is usually in the opposite direction, with perception preceding production (e.g., Shipley et al., 1969; Petretic and Tweney, 1977; Clark and Hecht, 1983). The question of why there is an asymmetry has been widely discussed, and there are two leading arguments. On the one hand, it is argued that children’s lexical representations are immature, and this has an impact on both their production and perception (e.g., Ferguson and Farwell, 1975; Vihman and Croft, 2007; Fikkert, 2010). These accounts explain the symmetry of developmental patterns in production and perception, but not the existence of a time-delay between the two. The second theoretical argument assumes that children’s lexical representations are adult-like, and inaccurate productions arise through articulatory limitations or difficulties in mapping representations to articulatory gestures (e.g., MacNeilage and Davis, 2000; Pierrehumbert, 2003; Inkelas and Rose, 2007). Results from the current study indicate that children’s representations are not adult-like, thus the second explanation cannot support our findings. Our data are more in line with a view that lexical representations are immature, and different behavior in each of the tasks results from the different tasks used and the different factors that affect behavior in production and perception tasks.

Production tasks, by nature, tap into children’s articulatory abilities. Whereas production is usually assumed to lag behind perception, in the phonological context tested here, ease of articulation may be helping participants and providing an inflated impression of children’s knowledge. This argument may be linked to the idea of phonological naturalness. Natural phonology argues that phonology is phonetically grounded, proposing a functional explanation for sounds or sound sequences embedded in ease of articulation and perception (Dressler, 1984; Westbury and Keating, 1986). According to this theory, voiceless segments in final position are natural, as are voiced segments following a nasal. Evidence is taken from articulatory and perceptual accounts (Hayes and Stivers, 1995; Solé, 2007), as well as typological prevalence (Locke, 1983; Pater, 1999) and ease of acquisition (Smith, 1973, 1979). If post-nasal voicing is more natural, it is likely that children will perceive it better and be able to produce it earlier. Therefore, a possible explanation for the results of Experiment 1 could be that children simply find it easier to produce a voiced segment in a post-sonorant context than a voiceless segment. This could also go some way to explaining children’s over-use of voicing in post-sonorant /t/ words. However, this cannot entirely explain the pattern of results because participants were, on the whole, producing a voiceless segment in plural words more often than a voiced segment, even in a post-sonorant context.

It could be argued that the prevalence and/or naturalness of post-nasal voicing affected adults’ coding of voicing in the children’s production tokens in Experiment 1. Despite attempts to remove lexical information from the tokens they were required to code by removing the onset and coda from the word, the crucial word-medial phonological context was maintained. Coders may have been biased to perceive voiced segments in a post-sonorant context, and have overestimated children’s ability to accurately produce voicing in these target words. Conducting a control test with adult speakers assessed this risk. Adult coders were highly reliable in determining the voicing of a medial obstruent in a VCV or VC_son._CV sequence in the absence of lexical information when spoken by an adult, thus indicating that they were able to overcome potential perceptual biases when coding tokens.

The final possible task-related difference is the manner in which responses were measured. In Experiment 1 children had to make a decision between [t] or [d], whereas in Experiment 2 they had to decide whether they found [t] or [d] more acceptable. Experiment 2 thus allowed for more gradient behavior in deciding which version is more or less acceptable, permitting the option of not making a decision, whereas Experiment 1 forced them to make a categorical decision. One way of addressing this would be to test children’s sensitivity to mispronunciations in a forced-choice task, requiring them to indicate whether a pronunciation is correct or not.

Data presented in this study indicate that children’s representations of voicing alternations are not adult-like. At 3 years of age, Dutch toddlers do not have reliable representations of which familiar stem forms in their lexicon require an alternation in the morphological paradigm, but they are aware that voicing alternations occur. In a task where no categorical decision is required their doubt or uncertainty becomes apparent. In a task that requires them to make a categorical decision, their behavior is subject to more subtle influences, including ease of articulation, but also the statistical distribution of phonological sequences.

These findings have implications for our understanding of how lexical representations develop in the mental lexicon. Our data do not support a model which assumes that the mental lexicon is purely exemplar-based (e.g., Bybee, 2001; Tomasello, 2003), and that toddlers store words in the form that they are heard. If this were the case we would expect higher production accuracy in Experiment 1, and greater sensitivity to mispronunciations in Experiment 2. Even for items that are expected to be familiar to children, our data suggest that children are computing complex forms online during speech production and perception. Our data speak in favor of a developmental course that allows for restructuring of the mental lexicon, such as proposed by Peperkamp and Dupoux (2002), Tesar and Prince (2003), or Vihman (2014). Although, the details of these theories differ, they are agreed in the assumption that infants first use bottom-up processing mechanisms to establish representations, and later are able to posit comparisons across items in their lexicon. In the case of Dutch-learning infants, they would first use phonotactic distributions to infer that there is no voicing contrast in final position in their language, and in the absence of any other evidence they will establish a lexical representation identical to the surface form. Once they are able to draw comparisons across morphological variants of the same lexical item they will notice which paradigms contain a voicing alternation and alter their representations as necessary. The 3-year-olds tested in this study are in the process of drawing comparisons across forms and restructuring their lexicon. They are aware that alternations occur, though they do not yet know specifically which lexical items require an alternation and which do not. In the absence of precise knowledge they are forced to rely on whichever cues are available to them, and their behavior varies depending on the nature of the task. One cue that children are able to make use of is the distributional statistics of the language. Other factors may include the functional load of voicing in the language, or the frequency of occurrence of voicing alternations (Buckler and Fikkert, 2015). Further work is needed to investigate the how the course of acquisition progresses, to identify additional factors that may influence the developmental trajectory and assess their individual contribution to development.

## Author Contributions

This work was completed in partial fulfillment of HB’s Ph.D. thesis. HB designed, carried out, executed, and wrote up the work under the supervision of PF.

## Conflict of Interest Statement

The authors declare that the research was conducted in the absence of any commercial or financial relationships that could be construed as a potential conflict of interest.

## Footnotes

1 Transcripts: CLPF corpus, all transcripts. Van Kampen corpus, laura01-laura41, sarah01-sarah34.

## References

[B1] Baayen R. H. Piepenbrock R. Van Rijn H. (1993). *The CELEX Lexical Database (CD-ROM).* Philadelphia: University of Pennsylvania, Linguistic Data Consortium.

[B2] Barr D. J. Levy R. Scheepers C. Tily H. J. (2013). Random effects structure for confirmatory hypothesis testing: keep it maximal. *J. Mem. Lang.* 68 255–278. 10.1016/j.jml.2012.11.001 PMC3881361 24403724

[B3] Bates D. Maechler M. Bolker B. (2015). *Package “lme4.”* Available at: http://lme4.r-forge.r-project.org/

[B4] Berman R. A. (1981). Regularity vs anomaly: the acquisition of Hebrew inflectional morphology. *J. Child Lang.* 8 265–282. 10.1017/S0305000900003184 7251706

[B5] Bittner D. Köpke K.-M. (2001). On the acquisition of German plural markings. *ZAS Papers Linguist.* 21 21–32.

[B6] Boersma P. Weenink D. (2011). *Praat: Doing Phonetics* by Computer version 5.2.33. Available at: http://www.praat.org/

[B7] Booij G. (1995). *The Phonology of Dutch.* Oxford: Clarendon Press.

[B8] Buckler H. (2014). *The Acquisition of Morphophonological Alternations Across Languages.* Nijmegen: Radboud University.

[B9] Buckler H. Fikkert P. (2015). Dutch and german 3-year-olds’ representations of voicing alternations. *Lang. Speech* 10.1177/0023830915587038 [Epub ahead of print]. 27363255

[B10] Bybee J. (2001). *Phonology and Language Use.* Cambridge: Cambridge University Press.

[B11] Cazden C. (1968). The acquisition of noun and verb inflections. *Child Dev.* 39 433–448. 10.2307/1126956 5649958

[B12] Clark E. V. Hecht B. F. (1983). Comprehension, production, and language acquisition. *Annu. Rev. Psychol.* 34 325–349. 10.1146/annurev.ps.34.020183.001545

[B13] Coady J. A. Aslin R. N. (2004). Young children’s sensitivity to probabilistic phonotactics in the developing lexicon. *J. Exp. Child Psychol.* 89 183–213. 10.1016/j.jecp.2004.07.004 15501451 PMC5531272

[B14] de Villiers J. de Villiers P. (1972). A cross-sectional study of the acquisition of grammatical morphemes in child speech. *J. Psycholinguist. Res.* 2 267–278. 10.1007/BF01067106 24197869

[B15] Dressler W. U. (1984). Explaining natural phonology. *Phonology* 1 29–51. 10.1017/S0952675700000282

[B16] Ernestus M. (2000). *Voice Assimilation and Segment Reduction in Casual Dutch: a Corpus-Based Study of the Phonology-Phonetics Interface.* Amsterdam: Free University.

[B17] Ernestus M. Baayen R. H. (2003). Predicting the unpredictable: interpreting neutralized segments in Dutch. *Language* 79 5–38. 10.1353/lan.2003.0076

[B18] Ernestus M. Baayen R. H. (2004). Analogical effects in regular past tense production in Dutch. *Linguistics* 42 873–903. 10.1515/ling.2004.031

[B19] Fenson L. Bates E. Dale P. S. Goodman J. C. Reznick J. S. Thal D. (1993). *The MacArthur Communicative Development Inventories: User’s Guide and Technical Manual.* Baltimore: Paul H. Brokes Publishing Co.

[B20] Ferguson C. A. Farwell C. B. (1975). Words and sounds in early language acquisition. *Language* 51 419–439. 10.2307/412864

[B21] Fikkert P. (1994). *On the Acquisition of Prosodic Structure.* Hague: Holland Academic Graphics.

[B22] Fikkert P. (2010). Developing representations and the emergence of phonology: evidence from perception and production. *Lab. Phonol.* 10 227–255.

[B23] Frisch S. A. Large N. R. Pisoni D. B. (2000). Perception of wordlikeness: effects of segment probability and length on the processing of non words. *J. Mem. Lang.* 42 481–496. 10.1006/jmla.1999.2692 21738287 PMC3129706

[B24] Ganong W. F. (1980). Phonetic categorization in auditory word perception. *J. Exp. Psychol.* 6 110–125. 10.1037/0096-1523.6.1.110 6444985

[B25] Golinkoff R. M. Hirsh-Pasek K. Cauley K. M. Gordon L. (1987). The eyes have it: lexical and syntactic comprehension in a new paradigm. *J. Child Lang.* 14 23–45. 10.1017/S030500090001271X 3558524

[B26] Hayes B. P. Stivers T. (1995). *Postnasal Voicing.* Los Angeles: University of California.

[B27] Hollich G. Jusczyk P. W. Luce P. A. (2002). “Lexical neighborhood effects in 17-month-old word learning,” in *Proceedings of the 26th Annual Boston University Conference on Language Development* (Boston, MA: Cascadilla Press) 314–323.

[B28] Hothorn T. Bretz F. Westfall P. (2008). Simultaneous inference in general parametric models. *Biometr. J.* 50 346–363. 10.1002/bimj.200810425 18481363

[B29] Inkelas S. Rose Y. (2007). Positional neutralization: a case study from child language. *Language* 83 707–736. 10.1353/lan.2008.0000

[B30] Jolly H. R. Plunkett K. (2008). Inflectional bootstrapping in 2-year-olds. *Lang. Speech* 51 45–59. 10.1177/00238309080510010401 18561543

[B31] Jusczyk P. W. Friederici A. D. Wessels J. M. Svenkerud V. Jusczyk A. M. (1993). Infants’ sensitivity to the sound patterns of native language words. *J. Mem. Lang.* 32 402–420. 10.1006/jmla.1993.1022

[B32] Jusczyk P. W. Luce P. A. Charles-Luce J. (1994). Infants’ sensitivity to phonotactic patterns in the native language. *J. Mem. Lang.* 33 630–645. 10.1006/jmla.1994.1030

[B33] Kager R. (1999). *Optimality Theory.* Cambridge: Cambridge University Press.

[B34] Kerkhoff A. (2007). *The Acquisition of Morpho-Phonology: The Dutch Voicing Alternation.* Utrecht: Utrecht University.

[B35] Kerkhoff A. De Bree E. H. (2005). “Acquisition of morpho-phonology in children with specific language impairment and typically developing children,” in *UiL-OTS Yearbook 2004* eds Kerkhoff A. de Lange J. Sadeh Leicht L. (Utrecht: LOT) 37–51.

[B36] Kouider S. Halberda J. Wood J. Carey S. (2006). Acquisition of English number marking: the singular-plural distinction. *Lang. Learn. Dev.* 2 1–25. 10.1207/s15473341lld0201_1

[B37] Kuijpers C. T. L. (1993). Temporal aspects of the voiced-voiceless distinction in speech development of young Dutch children. *J. Phonet.* 21 313–327.

[B38] Levelt C. C. (1994). *On the Acquisition of Place.* Hague: Holland Academic Graphics.

[B39] Locke J. L. (1983). *Phonological Acquisition and Change.* New York, NY: Academic Press.

[B40] MacNeilage P. F. Davis B. L. (2000). On the origin of internal structure of word forms. *Science* 288 527–531. 10.1126/science.288.5465.527 10775113

[B41] MacWhinney B. (2000). *The CHILDES Project: Tools for Analyzing Talk* Third Edn Mahwah, NJ: Lawrence Erlbaum Associates.

[B42] Mattys S. L. Jusczyk P. W. (2001). Phonotactic cues for segmentation of fluent speech by infants. *Cognition* 78 91–121. 10.1016/S0010-0277(00)00109-8 11074247

[B43] Mattys S. L. Jusczyk P. W. Luce P. A. Morgan J. L. (1999). Phonotactic and prosodic effects on word segmentation in infants. *Cogn. Psychol.* 38 465–494. 10.1006/cogp.1999.0721 10334878

[B44] Mervis C. B. Johnson K. E. (1991). Acquisition of the plural morpheme: a case study. *Dev. Psychol.* 27 222–235. 10.1037/0012-1649.27.2.222

[B45] Mirman D. Dixon J. A. Magnuson J. S. (2008). Statistical and computational models of the visual world paradigm: growth curves and individual differences. *J. Mem. Lang.* 59 475–494. 10.1016/j.jml.2007.11.006 19060958 PMC2593828

[B46] Munson B. (2001). Phonological pattern frequency and speech production in adults and children. *J. Speech Lang. Hear Res.* 44 778–792. 10.1044/1092-4388(2001/061) 11521771

[B47] Neijt A. Schreuder R. (2007). Asymmetrical phoneme-grapheme mapping of coronal plosives in Dutch. *Writt. Lang. Literacy* 10 219–234. 10.1075/wll.10.2.04nei

[B48] Park T.-Z. (1978). Plurals in child speech. *J. Child Lang.* 5 237–250. 10.1017/S0305000900007443

[B49] Pater J. (1999). “Austronesian nasal substitution and other NC effects,” in *The Prosody-Morphology Interface* eds Kager R. van der Hulst H. Zonneveld W. (Cambridge: Cambridge University Press) 310–343.

[B50] Peperkamp S. Dupoux E. (2002). “Coping with phonological variation in early lexical acquisition,” in *The Process of Language Acquisition* ed. Lasser I. (Berlin: Peter Lang Verlag) 359–385.

[B51] Petretic P. A. Tweney R. D. (1977). Does comprehension precede production? The development of children’s responses to telegraphic sentences of varying grammatical adequacy. *J. Child Lang.* 4 201–209. 10.1017/S0305000900001604

[B52] Pierrehumbert J. B. (2003). Phonetic diversity, statistical learning, and acquisition of phonology. *Lang. Speech* 46 115–154. 10.1177/00238309030460020501 14748442

[B53] R Core Team. (2012). *R: A Language and Environment for Statistical Computing.* Vienna: R Foundation for Statistical Computing.

[B54] Ravid D. Schiff R. (2009). Morphophonological categories of noun plurals in Hebrew: a developmental study. *Linguistics* 47 45–63. 10.1515/LING.2009.002

[B55] Raymond W. D. Healy A. F. McDonnel S. Healy C. A. (2008). Acquisition of morphological variation: the case of the English definite article. *Lang. Cogn. Process.* 24 89–119. 10.1080/01690960802075489

[B56] Reetz H. (2010). *Reelex - Reetz-CELEX Interface.* Available at: http://web.phonetik.uni-frankfurt.de/simplex.html

[B57] Schaerlaekens A. M. (1977). *De Taalontwikkeling Van Het Kind: Een Oriëntatie in Het Nederlandstalig Onderzoek.* Groningen: Wolters-Noordhoff.

[B58] Seidl A. H. Cristia A. Bernard A. Onishi K. H. (2009). Allophonic and phonemic contrasts in infants’ learning of sound patterns. *Lang. Learn. Dev.* 5 191–202. 10.1080/15475440902754326

[B59] Shipley E. F. Smith C. S. Gleitman L. R. (1969). A study in the acquisition of language: free responses to commands. *Language* 45 322–342. 10.2307/411663

[B60] Singer J. D. Willett J. B. (2003). *Applied Longitudinal Data Analysis.* Oxford: Oxford University Press.

[B61] Slis I. Cohen A. (1969). On the complex regulating the voiced-voiceless distinction I. *Lang. Speech* 12 80–102. 10.1177/002383096901200301 5792375

[B62] Smith B. L. (1978). Temporal aspects of English speech production: a developmental perspective. *J. Phonet.* 6 37–67.

[B63] Smith B. L. (1979). A phonetic analysis of consonantal devoicing in children’s speech. *J. Child Lang.* 6 19–28. 10.1017/S0305000900007595

[B64] Smith B. L. Sugarman M. D. Long S. H. (1983). Experimental manipulation of speaking rate for studying temporal variability in children’s speech. *J. Acoust. Soc. Am.* 74 744–749. 10.1121/1.389860 6630730

[B65] Smith N. V. (1973). *The Acquisition of Phonology: A Case Study.* Cambridge: Cambridge University Press.

[B66] Solé M.-J. (2007). “Compatibility of features and phonetic content. The case of nasalization,” in *Proceedings of ICPhS 2007* Saarbrücken 261–266.

[B67] Storkel H. L. (2001). Learning new words: phonotactic probability in language development. *J. Speech Lang. Hear. Res.* 44 1321–1337. 10.1044/1092-4388(2001/103) 11776368

[B68] Storkel H. L. Rogers M. A. (2000). The effect of probabilistic phonotactics on lexical acquisition. *Clin. Linguist. Phonet.* 14 407–425. 10.1080/026992000415859

[B69] Swingley D. Aslin R. N. (2000). Spoken word recognition and lexical representation in very young children. *Cognition* 76 147–166. 10.1016/S0010-0277(00)00081-0 10856741

[B70] Tesar B. Prince A. (2003). “Using phonotactics to learn phonological alternations,” in *Proceedings from the Annual Meeting of the Chicago Linguistic Society* Vol. 39 (Chicago: Chicago Linguistic Society) 241–269.

[B71] Tobii Technology (2008). *Tobii Studio 1.X User Manual.* Daneryd: Tobii Technology.

[B72] Tomasello M. (2003). *Constructing a Language: A Usage-Based Theory of Language Acquisition.* Cambridge, MA: Harvard University Press.

[B73] Treiman R. Kessler B. Knewasser S. Tincoff R. Bowman M. (2000). “English speakers’ sensitivity to phonotactic patterns,” in *Papers in Laboratory Phonology V: Acquisition and the Lexicon* eds Broe M. B. Pierrehumbert J. B. (Cambridge: Cambridge Scholar Press) 269–282.

[B74] van de Velde H. Gerritsen M. van Hout R. (1996). The devoicing of fricatives in Standard Dutch: a real-time study based on radio recordings. *Lang. Variat. Change* 8 149–175. 10.1017/S0954394500001125

[B75] Van de Vijver R. Baer-Henney D. (2011). “Acquisition of voicing and vowel alternations in German,” in *Proceedings of the 35th Annual Boston University Conference on Language Development* eds Danis N. Mesh K. Sung H. (Somerville, MA: Cascadilla Press) 603–615.

[B76] Van Kampen J. (1994). The learnability of the left branch condition. *Linguistics* 83–94. 10.1075/avt.11.10kam

[B77] Van Wijk J. (2007). *The Acquisition of the Dutch Plural.* Utrecht: Utrecht University.

[B78] Vihman M. M. (2014). *Phonological Development: The First Two Years* 2nd Edn Oxford: Wiley-Blackwell.

[B79] Vihman M. M. Croft W. (2007). Phonological development: toward a “radical” templatic phonology. *Linguistics* 45 683–725. 10.1515/LING.2007.021

[B80] Vitevitch M. S. Luce P. A. Charles-Luce J. Kemmerer D. (1997). Phonotactics and syllable stress: Implications for the processing of spoken nonsense words. *Lang. Speech* 40 47–62. 10.1177/002383099704000103 9230698

[B81] Westbury J. R. Keating P. A. (1986). On the naturalness of stop consonant voicing. *J. Linguist.* 22 145–166. 10.1017/S0022226700010598

[B82] White K. S. Peperkamp S. Kirk C. J. Morgan J. L. (2008). Rapid acquisition of phonological alternations by infants. *Cognition* 107 238–265. 10.1016/j.cognition.2007.11.012 18191826 PMC2941201

[B83] Zamuner T. S. Gerken L. Hammond M. (2004). Phonotactic probabilities in young children’s speech production. *J. Child Lang.* 31 515–536. 10.1017/S0305000904006233 15612388

[B84] Zamuner T. S. Kerkhoff A. Fikkert P. (2011). Phonotactics and morphophonology in early child language: evidence from Dutch. *Appl. Psychol.* 33 481–499. 10.1017/S0142716411000440

[B85] Zink I. Lejaegere M. (2002). *N-CDIs: Lijsten Voor Communicatieve Ontwikkeling. Aanpassing en Hernormering van deMacArthur CDIs van Fenson et al. [A CDI user’s Manual with Normative and Validity Data].* Leusden: Acco.

[B86] Zonneveld W. (2004). De verwerving van een morfologisch proces: nederlandse meervoudsvorming. *Nederlandse Taalkunde* 9 1–28.

